# Extracellular Vesicle Heterogeneity: Subpopulations, Isolation Techniques, and Diverse Functions in Cancer Progression

**DOI:** 10.3389/fimmu.2018.00738

**Published:** 2018-04-30

**Authors:** Eduard Willms, Carlos Cabañas, Imre Mäger, Matthew J. A. Wood, Pieter Vader

**Affiliations:** ^1^Department of Physiology, Anatomy and Genetics, University of Oxford, Oxford, United Kingdom; ^2^Centro de Biología Molecular Severo Ochoa (CSIC-UAM), Madrid, Spain; ^3^Department of Microbiology I (Immunology), Faculty of Medicine, Universidad Complutense, Madrid, Spain; ^4^Institute of Technology, University of Tartu, Tartu, Estonia; ^5^Department of Clinical Chemistry and Haematology, University Medical Center Utrecht, Utrecht, Netherlands; ^6^Department of Experimental Cardiology, University Medical Center Utrecht, Utrecht, Netherlands

**Keywords:** extracellular vesicles, exosomes, microvesicles, cancer, heterogeneity, subpopulations

## Abstract

Cells release membrane enclosed nano-sized vesicles termed extracellular vesicles (EVs) that function as mediators of intercellular communication by transferring biological information between cells. Tumor-derived EVs have emerged as important mediators in cancer development and progression, mainly through transfer of their bioactive content which can include oncoproteins, oncogenes, chemokine receptors, as well as soluble factors, transcripts of proteins and miRNAs involved in angiogenesis or inflammation. This transfer has been shown to influence the metastatic behavior of primary tumors. Moreover, tumor-derived EVs have been shown to influence distant cellular niches, establishing favorable microenvironments that support growth of disseminated cancer cells upon their arrival at these pre-metastatic niches. It is generally accepted that cells release a number of major EV populations with distinct biophysical properties and biological functions. Exosomes, microvesicles, and apoptotic bodies are EV populations most widely studied and characterized. They are discriminated based primarily on their intracellular origin. However, increasing evidence suggests that even within these EV populations various subpopulations may exist. This heterogeneity introduces an extra level of complexity in the study of EV biology and function. For example, EV subpopulations could have unique roles in the intricate biological processes underlying cancer biology. Here, we discuss current knowledge regarding the role of subpopulations of EVs in cancer development and progression and highlight the relevance of EV heterogeneity. The position of tetraspanins and integrins therein will be highlighted. Since addressing EV heterogeneity has become essential for the EV field, current and novel techniques for isolating EV subpopulations will also be discussed. Further dissection of EV heterogeneity will advance our understanding of the critical roles of EVs in health and disease.

## Introduction

Extracellular vesicles (EVs) are heterogeneous populations of naturally occurring nano to micro-sized membrane vesicles released by essentially all cell types. EVs are enclosed by a lipid bilayer and range in size from 30 to 10,000 nm in diameter. They have emerged as a novel and important player in intercellular communication, mainly through their ability to transfer their biological content, consisting of proteins, lipids, and nucleic acids, to recipient cells (1–3). It is increasingly evident that EVs play a major role in the regulation of physiological processes, such as tissue repair (4), stem cell maintenance (5), and coagulation (6). In terms of pathophysiological processes, EVs have established as important players in diseases such as cancer (7), neurodegenerative disease (8), and viral infection (9).

### Discovery and Study of EVs

Reports on the occurrence of what we now call EVs were first published in the late 1960s, with researchers referring to observed extracellular structures or lipid-rich particles as “platelet-dust” or “matrix-vesicles” (10, 11). It took approximately another 10 years for researchers to start reporting the presence of “microparticles” and “microvesicles” released by cells (12, 13). The term exosomes (EXOs) emerged in 1981 (14), together with a basic understanding regarding the underlying intracellular biogenesis pathways being demonstrated by the groups of Johnstone and Stahl (15, 16).

The interest in EVs has since then grown rapidly, with efforts being made to understand EV biology, biological functions, and their application as therapeutics and biomarkers. The ability of EVs to transfer their cellular content from donor to recipient cell seems to be their most interesting characteristic for potential applications. First, the packaged content reflects the state of the donor cell, which renders them useful as biomarkers (17). Moreover, this intrinsic characteristic makes them interesting candidates for intracellular delivery of therapeutics (18). Remarkably, EXOs have become the most widely studied EV population, which perhaps falsely portrays them as more important and more interesting than other EV populations (19), an assumption which is not supported by current evidence regarding precise biological functions of other secreted EV populations.

Our understanding of the fundamental role of EVs in multiple physiological and pathophysiological processes is constantly increasing. Perhaps most widely described is their role in cancer, affecting tumor genesis and progression, clearly highlighting their relevance in disease (7).

### Roles of EVs in Cancer

In recent years, it has become evident that the tumor microenvironment plays a pivotal role in cancer (20). EVs seem to be involved in influencing both the tumor microenvironment directly surrounding the primary tumor and microenvironments at distant sites which facilitates subsequent successful metastasis of disseminated cancer cells. In that regard, EVs have been shown to affect multiple underlying key processes, including oncogenic transfer, angiogenesis, immune modulation, thrombosis, and pre-metastatic niche formation (21–26).

Tumor genesis involves occurrence and accumulation of genetic alterations. EVs have been shown to be capable of transferring their oncogenic cargo, and as such induce metastatic traits leading to enhanced tumorigenesis. For example, tumor-derived EVs can confer transformed traits of cancer cells (e.g., anchorage-independent growth and enhanced survival capability) on recipient fibroblasts and endothelial cells, through cotransfer of the protein cross-linking enzyme tissue transglutaminase and the extracellular matrix protein fibronectin (FN) (21).

Similarly, the transfer of mutant KRAS proto-oncogene *via* EVs has been shown to increase the growth of wild-type KRAS-expressing recipient colon cancer cells (27). Furthermore, EVs derived from metastatic melanoma cells are capable of promoting a metastatic phenotype *via* activation of mitogen-activated protein kinase pathways in non-metastatic recipient melanoma cells (22).

Tumor growth beyond microscopic size is dependent on adequate nutrient and oxygen supply, a process which is ultimately dependent on increased angiogenesis. EVs derived from hypoxic glioma cells are capable of influencing the surrounding vasculature by induction of pro-angiogenic processes (23). It has also been shown that EVs derived from A431 squamous carcinoma cells can directly transfer oncogenic epidermal growth factor receptor (EGFR) to endothelial cells, leading to induction of angiogenesis (28).

Tumor-derived EVs seem to play an important role in coagulation and the development of thrombosis in cancer patients, putting cancer patients at risk of increased morbidity and mortality due to thrombotic events (24). Tissue factor (TF) and P-selectin glycoprotein ligand-1 (PSGL-1) expressed on tumor-derived EVs have been shown to play an important role in cancer-associated thrombosis (29). In a mouse model for deep venous thrombosis, pancreatic tumor-bearing mice showed an increase in thrombus formation compared with mice without cancer (30). The observed increase was found to be mediated by TF bearing EVs.

In addition to the aforementioned roles of EVs in the transfer of oncogenic traits and promotion of angiogenesis, EVs are capable of influencing multiple aspects of the immune system (31–33). A number of findings published by the Whiteside group clearly highlight the important role of cancer-derived EVs in immunomodulation. Apoptosis of effector cytotoxic T cells, observed in the peripheral circulation of patients with oral squamous cell carcinoma, was found to be mediated through FasL (Fas ligand) positive EVs (34). Furthermore, cancer-derived EVs were found to exert a number of effects on T regulatory cells (Tregs), such as induction of Treg, promotion Treg expansion, upregulation of Treg suppressor function, and enhancement of Treg resistance to apoptosis (35). These effects provide a mechanism for regulation of peripheral tolerance by tumors and support immune evasion of cancer.

Immunosuppression is considered as one of the key factors stimulating tumor progression, with myeloid-derived suppressor cells (MDSCs) being one of the most important players mediating this process (36). It has been demonstrated that tumor-derived EVs are able to promote T cell-dependent immunosuppressive functions of MDSCs, an effect which was found to be mediated through heat-shock protein 72 (Hsp72) expressed on the EV surface. Binding of Hsp72 to TLR2 on MDSCs leads to promotion of MDSCs suppressive properties, *via* activation of STAT3 activation (25). Similarly, breast cancer-derived EVs containing prostaglandin 2 and transforming growth factor beta have been shown to mediate immunosuppressive functions through influencing MDSCs (37). Cancer-derived EVs have also been shown to influence stromal fibroblasts, ultimately leading to stimulation of tumor growth, metastasis, and therapy resistance (38). This process is orchestrated through an intricate signaling process between cancer and stromal cells, with transfer of unshielded RNA (RN7SL1) being crucial.

Recent findings shed light on the important role of the immunosuppressive molecule programmed cell death ligand 1 (PD-L1) present on EVs. Glioblastoma-derived EVs were found to directly bind to programmed cell death protein-1 (PD-1), ultimately leading to immune evasion through inhibition of T cell activation and proliferation (39).

The functional role of tumor-derived EVs in cancer metastasis is perhaps the most well established and explored role of EVs in cancer. Metastasis of tumor cells is a pivotal process in cancer progression. As postulated over a century ago by Stephen Paget in his Seed and Soil hypothesis, successful metastasis is dependent on the interaction between the receiving cells or organ (i.e., “the soil”) and the circulating tumor cell (i.e., “the seed”) (40). As outlined below, EVs play a prominent role in this process, affecting “the soil” at distant sites, and stimulating the formation of a so-called pre-metastatic niche which favors the outgrowth of disseminated tumor cells. Second, EVs are capable of changing the metastatic behavior of cancer cells (i.e., “the seed”). Findings by Hood et al. demonstrate that melanoma-derived EVs are able to home to sentinel lymph nodes, leading to recruitment of disseminated melanoma cells, and stimulation of metastatic factors involved in angiogenesis and extracellular matrix remodeling (41). Furthermore, melanoma EVs have been shown to affect bone marrow-derived cells (BMDCs), which are essential for the generation of a tumor microenvironment favoring metastasis (42). Transfer of EV-packaged MET was found to be the key driver of these effects, resulting in enhanced mobilization of BMDCs. Remarkably, preconditioning with melanoma-derived EVs led to an increase in the metastatic tumor burden and distribution in target tissues, even for tumors of different origins with a low-metastatic capacity (42).

It has also been shown that pancreatic tumor-derived EVs are taken up by Kupffer cells in the liver, which leads to recruitment of bone marrow-derived macrophages through increased FN production by hepatic stellate cells (26). Migration inhibitory factor, a protein enriched in pancreatic EVs, was identified as a key protein in mediating this process. Aforementioned findings all highlight the effects of cancer-derived EVs on recipient cells, and their important role in metastasis.

Tumor cells release increased numbers of EVs into their extracellular environment compared with healthy cells and are exposed to large quantities of EVs secreted by various cell types. As a result, precisely identifying which EVs mediate functional effects on recipient cells, and which recipient cell have successfully taken up EVs, remains complicated. To study EV-mediated exchange, Zomer et al. developed a Cre-LoxP-based system which allows for intravital imaging of successful EV transfer (43). This system allowed for elucidation of EV-mediated exchange of metastatic traits, showing that breast cancer cells with low metastatic capacity (T47D cells) show increased metastatic potential after uptake of EVs derived from highly metastatic breast cancer cells (MDA-MB-231 cells) *in vivo*.

Aforementioned findings clearly highlight multiple roles of EVs in cancer. However, the heterogeneous nature of tumor cells, and heterogeneity within secreted EVs adds another layer of complexity that remains to be addressed.

### Challenge for the Field—EV Heterogeneity

Cells release large numbers of EVs into their extracellular environment, which exert diverse biological effects on recipient cells. Diverse effects reported for EVs indicate that either each EV may exert multiple functions, perhaps depending on secondary conditions, or cells release populations of EVs with unique functions. For example, analyses of miRNA stoichiometry in EVs reveal that most of the EVs released by cells will not contain biologically significant numbers of miRNA copies (44). There is, however, increasing evidence for functional transfer of miRNAs *via* EVs, which seems to be a highly selective and infrequent event (45–47). These findings indicate the potential existence of EV subpopulations with unique characteristics and miRNA content. Findings have confirmed that differential packaging of miRNAs into distinct subpopulations of EVs released by tumor cells occurs (48). Remarkably, the potential of heterogeneity existing within released EVs was already raised by Johnstone et al. in their 1987 paper (15), stating that “It is also as yet unclear whether each EXO contains a mixture of all externalized components or if a mixed population is externalized.”

The EV field has made great progress defining major EV populations based on their underlying biogenesis, and biophysical characteristics, mainly as a result of improving technologies for isolation and characterization, and increased understanding of EV content and biology. Lack of guidelines regarding nomenclature of EVs has, however, resulted in an eclectic mix of names being published, for what are in essence overlapping populations (49). One of the biggest challenges for the EV field at this stage is addressing heterogeneity of secreted EVs, and heterogeneity within EV populations. Shedding light on the diversity will allow for a better understanding of the precise role of EVs in both physiological and pathophysiological processes, ultimately accelerating development of EVs as therapeutics and diagnostics.

## EVs and EV Heterogeneity

**Figure 1:**
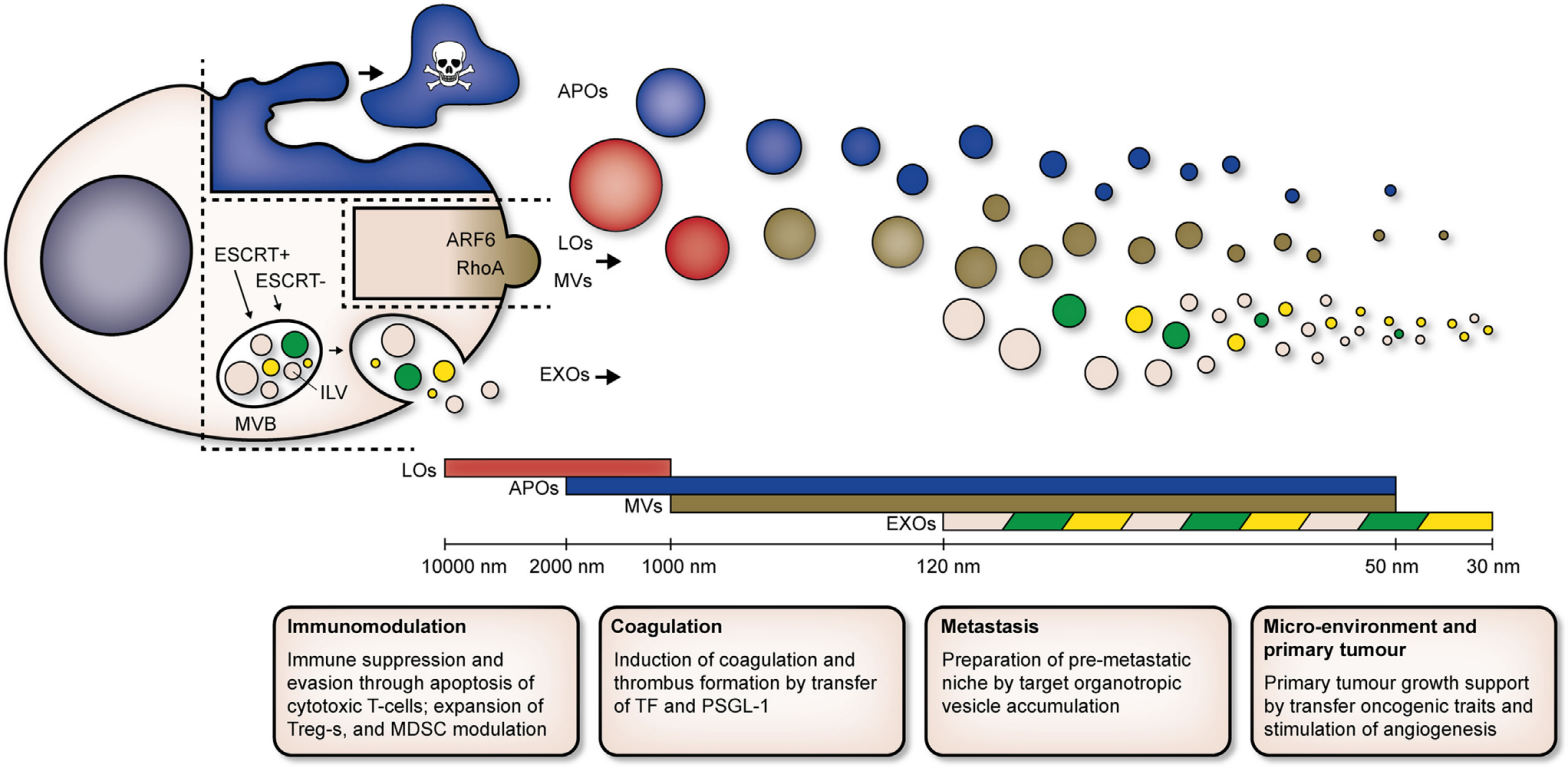
Cells release heterogeneous populations of EVs with overlapping sizes. APOs (blue) are released by cells undergoing apoptosis. LOs (red) and MVs (brown) are derived directly from the plasma membrane, ARF6 and RhoA are key players in biogenesis of MVs. EXOs (pink) are derived from intracellular endosomal compartments. ILVs form within MVBs and are subsequently released upon fusion of MVBs with the plasma membrane. Both ESCRT-dependent (ESCRT+) and -independent (ESCRT−) pathways are involved in biogenesis of EXOs. Unique subpopulations of EXOs (as indicated by green and yellow EVs) have been identified. Abbreviations: MVB, multivesicular body; ILV, intraluminal vesicle; ESCRT+, endosomal sorting complex required for transport-dependent; ESCRT, endosomal sorting complex required for transport-independent; ARF6, ADP-ribosylation factor 6; RhoA, Ras homolog gene family, member A; EVs, extracellular vesicles; APOs, apoptotic bodies; LOs, large oncosomes; MVs, microvesicles; EXOs, exosomes; Tregs, T regulatory T cells; MDSCs, myeloid-derived suppressor cells; TF, tissue factor; PSGL-1, P-selectin glycoprotein ligand-1.

To date, it seems that EVs can be classified into three main populations: EXOs, microvesicles (MVs), and apoptotic bodies (APOs). EV populations which are unique to a disease, harnessing unique biophysical characteristics and compositions, have been identified as well [e.g., large oncosomes (LOs) in cancer]. Current classification of EV populations is based on the mechanisms of biogenesis of each population, as well as their biophysical properties (Figure 1).

### EXOs, MVs, and APOs

Exosomes are derived from intracellular endosomal compartments. Their formation occurs through the endolysosomal pathway, in which EXOs form as intraluminal vesicles (ILVs) within multivesicular bodies (MVBs). They are subsequently released by cells upon fusion of MVBs with the plasma membrane. In contrast to other EV populations, EXOs exhibit a relatively homogenous size distribution, ranging from 30 to 120 nm in diameter.

Exosome biogenesis depends on the endosomal sorting complex required for transport (ESCRT) machinery that is responsible for protein sorting and ILV formation (50, 51). This machinery is composed of more than 20 proteins assembled into 4 protein complexes (ESCRT-0, -I, -II, and -III) and associated proteins [e.g., vacuolar protein sorting-associated protein 4, ALG-2 interacting protein X (ALIX)]. The precise implication of each of these ESCRT complexes and associated proteins in ILV formation has been reviewed elsewhere (52).

A comprehensive RNA interference screen, targeting individual components of the ESCRT machinery, highlighted that alterations in this machinery can result in EV heterogeneity both in size and in composition. Colombo et al. evaluated effects on secretion of EXOs (100,000 × *g* pellets) by HeLa-CIITA-OVA and dendritic cells (DCs) (53). Silencing of ESCRT-0 [hepatocyte growth factor-regulated tyrosine kinase substrate (HRS) and signal transducing adapter molecule 1 (STAM1)] and ESCRT-1 (tumor susceptibility gene 101 protein TSG101) components decreased secretion of exosomal proteins (CD63, MHC II). Silencing of HRS mainly resulted in a decrease of EXOs with a size between 50 and 200 nm, indicating that HRS is not involved in the secretion of EXOs smaller than 50 nm. On the contrary, STAM1 and ALIX seemed to play a role in the secretion of EXOs smaller than 50 nm. The authors also observed changes in EXO composition, e.g., a reduction of exosomal CD63 and MHC II after depletion of TSG101 and STAM1. Interestingly, quantification of the expression of CD63 and MHC II indicated that only 20% of the total EXOs released by cells express both markers. This finding clearly demonstrates the heterogeneous nature of secreted EXOs.

Multivesicular bodies and EXOs can also form in the absence of ESCRT machinery (54), and tetraspanins seem to be particularly important in this ESCRT-independent mechanism of ILV and EXO biogenesis. CD63 directly participates in ESCRT-independent protein sorting and ILV formation in melanocytes, although EXO secretion was not assessed (55, 56). EXO secretion is defective in DCs from CD9 knockout mice, while overexpression of CD9 or CD82 in human embryonic kidney 293 cells increases the incorporation of β-catenin in EXOs (57).

The sphingolipid ceramide has been shown to play a role in EXO biogenesis as well (58), with inhibition of neutral sphingomyelinases [responsible for breaking sphingomyelin (SM) down into phosphocholine and ceramide] resulting in a reduction of EXO release (53, 54, 58). This ceramide-dependent biogenesis constitutes yet another biogenesis pathway not dependent on ESCRT machinery. Interestingly, a competitive relationship between aforementioned ESCRT-dependent and -independent mechanism seems to exist, which influences formation of differently sized ILV subpopulations within MVBs (59). Based on these findings, it is tempting to speculate that these competitive relationships ultimately contribute to heterogeneity.

Other essential players in EXO biogenesis and intracellular vesicle trafficking in general are Rab proteins, which comprise the largest part of the Ras-like small GTPase superfamily [reviewed in Ref. (60, 61)]. There is strong evidence for the role of a number of specific Rab proteins in EXO release. For instance, it has been shown that Rab11 knockdown inhibits the release of transferrin receptor- and heat-shock protein 70 kDa-expressing EXOs (62) in a chronic myeloid leukemia cell line. Frühbeis et al. have shown that silencing of Rab35 results in inhibition of release of proteolipid protein- and ALIX-expressing EXOs (63) from primary oligodendrocytes. Furthermore, in HeLa cells, inhibition of Rab27 results in a decrease of CD63-, CD81- and MHC II-expressing EXOs (64). The roles of Rab27a and Rab27b in EXO release are well defined and have been confirmed in different cell types by multiple groups (42, 61, 64). Most importantly, knockdown of Rab27a in melanoma cells has been shown to decrease EXO production and inhibit tumor growth and metastasis in mice (42). A more elaborate review on the exact functions of Rab proteins involved in EV biogenesis, secretion, and intercellular interactions has been given elsewhere (53).

In contrast to EXOs, MVs represent a population of non-apoptotic EVs which originate directly from the plasma membrane. Throughout literature, numerous different names including shedding vesicles, ectosomes, and microparticles have been used to refer to MVs. MVs range in size from 50 to 1,000 nm in diameter. The biogenesis and release of MVs from the plasma membrane is dependent on a number of processes, reviewed elsewhere (65). Briefly, when cell activating signals lead to an increase of cytosolic Ca^2+^ levels, this increase ultimately leads to changes in transbilayer lipid distribution and membrane blebbing, through alterations in the activity of the enzymes flippase, translocase, and scramblase (65, 66).

One of the most well-described regulators of MV shedding is ADP-ribosylation factor 6 (ARF6), a member belonging to the Ras superfamily of small GTPases (67, 68). ATP-mediated activation of P2X receptors leading to rearrangements of the cell membrane seems to be involved in MV release as well (69). Regulation of actin dynamics and reorganization of the actin cytoskeleton through the small GTPase Ras homolog gene family member A (RhoA) and subsequent activation of associated kinase Rho-associated coiled coil-containing kinases are also involved in MV formation (70).

Finally, cells undergoing apoptosis release a population of EVs called APOs through outward blebbing and fragmentation of the cell membrane (71). APOs have a broad size range between 50 and 2,000 nm in diameter. Although their content is generally thought of as randomly packaged, there is evidence of sorting of RNA and DNA into separate distinct APO subpopulations (72).

### Large Oncosomes

Large oncosomes represent a population of EVs whose release seems to be unique to cancer cells (73). They represent yet another population of non-apoptotic EVs originating directly from the plasma membrane. The name LOs emphasizes their atypical large size (1–10 µm) and ability to transfer oncogenic material. They originate through membrane shedding from cancer cells with an amoeboid phenotype, a process which can be induced through overexpression of oncoproteins [e.g., myristoylated Akt1, heparin-binding epidermal growth factor, and caveolin-1 (Cav-1)], silencing of the cytoskeletal regulator Diaphanous-related formin-3, or activation of Akt1 and EGFR pathways. Importantly, their formation does not coincide with increased rates of cell death, further highlighting that LOs are a unique class of EVs with biogenesis distinct from APOs.

Large oncosomes are specifically enriched in a membrane-localized cytokeratin-18 and express only very low levels of tetraspanins CD9, CD63, and CD81. Cav-1 has been reported as a marker for LOs, and results suggest that Cav-1 positive LOs can be used to detect metastatic disease in patients with prostate cancer (74, 75). Evaluation of the miRNA content of LOs showed sorting of specific miRNA species into LOs versus smaller EV populations (75). Interestingly, the authors also report the presence of programmed cell death 6 interacting protein, also known as ALIX, in LOs, despite the fact that increasing evidence and in depth proteomics have highlighted ALIX as an EXO marker. Similar to MVs, LOs originate from the plasma membrane, and as a result, ARF6 is found in LOs as well. Overlaps in composition and biogenesis further highlight the complexity of obtaining, and characterizing, pure EV populations.

### Tetraspanins as Markers for EV Populations

Proteomic analyses have shown that different tetraspanins with wide cellular expression (CD9, CD63, CD81, and CD82) are highly enriched in EXOs relative to their content in the respective producing cells (76–78). Accordingly, these proteins have been considered as good general exosomal markers. However, caution should be exercised when considering tetraspanins as truly specific exosomal markers because these proteins are also abundantly expressed on the cell surface and thus become incorporated in other types of EVs that are generated by direct budding from the plasma membrane and do not have an endocytic origin. In this regard, different reports have shown that CD9, CD63, and CD81 are not only abundant in EXOs but also in MVs, complicating their usefulness for discrimination among these different types of EVs (56, 79). In a model of mouse mammary carcinoma cells, CD9 has been shown not to be specific for EXOs, as it is also present on larger vesicles whose secretion is not inhibited by Rab27a shRNA silencing (80). CD81 and CD63 have also been detected in both MVs and EXOs secreted by different types of cells (79).

Recently, a systematic categorization of EVs has been proposed based on a comprehensive comparative proteomic analysis of the EVs recovered after differential centrifugations followed by either flotation in sucrose or iodixanol gradients or immuno-isolation based on CD9, CD81, and CD63 antibodies (81). This categorization, which according to the authors could be applied to EVs from any source, including cell culture medium or any biological fluid, includes (i) large EVs pelleting at low centrifugation speed (2,000 × *g*); (ii) medium EVs pelleting at intermediate speed (10,000 × *g*); (iii) small EVs (sEVs) pelleting at high speed (100,000 × *g*). Among these sEVs, in turn, four subcategories were defined: (iiia) sEVs coenriched in CD63, CD9, and CD81 and in endosomal markers (*bona fide* EXOs); (iiib) sEVs devoid of CD63 and CD81 but enriched in CD9 (associated with plasma membrane and/or early endocytic signature); (iiic) sEVs devoid of CD63, CD81 and CD9; (iiid) sEVs enriched in extracellular proteins (ECM) or serum proteins, with the latter two subcategories not being associated with an endosomal origin, and therefore with true EXOs. In this study, several protein markers were also proposed for the identification of the different subpopulations of EVs. For instance, syntenin-1 and TSG101 were proposed to be specific for the tetraspanin-enriched EVs representing the *bona fide* EXOs in category iiia. Given the different composition and cellular origin of the distinct subpopulations of EVs, the functional effects previously attributed to “exosomes” on the basis of their tetraspanin-enrichment and size as the principal criteria for their identification should be considered cautiously. It is also apparent from this extensive proteomic analysis that EXOs produced by different cell types and even from the same cells may greatly differ qualitatively and quantitatively with regard to their specific tetraspanin content.

## Techniques for Isolation of EV Populations

Addressing EV heterogeneity has become more important for the EV field, isolation and purification methods have been optimized aiming to allow isolation of pure populations, and even identification of distinct subpopulations. An overview of commonly used methods, with a general description, and their advantages and disadvantages for isolation and identification of EV populations and subpopulations are listed below.

### Differential Ultracentrifugation (dUC) and Density Gradient Flotation (DG)

Ultracentrifugation remains the most commonly used technique for isolation of EVs from cell culture supernatant and other biofluids. Successive centrifugation at high centrifugal forces allows for elimination of dead cells and cellular debris and subsequent pelleting of EVs.

A detailed protocol for isolation of EV populations was published by Théry et al. in 2006 (82). In short, large EVs (e.g., LOs and APOs) are pelleted by centrifugation at 2,000 × *g*, MVs are pelleted by centrifugation at 10,000 × *g*, whereas EXOs are pelleted at centrifugal forces of 100,000 × *g* and above. Pelleted EVs are washed by resuspension and a subsequent second round of pelleting.

This protocol has been further optimized (i.e., changes were made to centrifugal force and centrifugation time) and utilized by many groups in the EV field for isolation of EV populations. Using such optimized protocols, TSG101 and CD63 were previously identified as marker proteins for EXOs (100,000 × *g* pellets) released by breast cancer cells (SK-BR3 and MCF-7 cells), whereas the protein plasma membrane-bound extracellular MMP inducer (EMMPRIN) was present exclusively on MVs (14,000 × *g* pellets) (83). Crescitelli et al. confirmed the successful isolation of populations on the basis of distinct RNA content of different populations of EVs derived from different cell lines (79). At the same time, when Jeppesen et al. thoroughly investigated the influence of a number of parameters (e.g., centrifugal force and time) on EXO yield and composition after dUC isolation (84), large differences between sedimentation patterns and efficiency of EXOs derived from different cell lines were observed. Interestingly, differential expression patterns of exosomal markers TSG101 and syntenin were also found. Syntenin was found to be enriched compared with TSG101 in EXOs isolated at high centrifugal force. This finding hints to the presence of EXO subpopulations with different composition.

Despite these interesting findings, it has since then been shown that yield and purity of UC-isolated EVs is greatly influenced by rotor type and centrifugation time (85). Furthermore, dUC does not allow for absolute separation of EV populations, as, although less efficiently, smaller EVs will also pellet at lower centrifugal forces and *vice versa*, resulting in heterogeneity within isolated populations, in turn making the study of EV subtypes using dUC only very difficult.

Density gradient flotation is a method commonly used in the EV field to increase purity of isolated EVs. It has mainly been employed for elimination of copurified non-EV material or EV fragments which arise during dUC. In DG, EVs are allowed to float into a gradient of increasing dilutions of viscous solutions (e.g., sucrose or iodixanol). Upon centrifugation, EVs migrate to their (equilibrium) density. The migration speed or rate of flotation depends on the size, shape, and density of the EV. DG has also been employed to address EV heterogeneity. Findings by Aalberts et al. highlighted the existence of populations of prostasomes in seminal plasma from vasectomized men (86). Prostasomes are small membrane vesicles secreted after fusion of multivesicular bodies with the plasma membrane of prostate epithelial cells. A wide variety of effects in seemingly unrelated biological processes have been reported for prostasomes, suggesting the likelihood of distinct subpopulations. Sucrose gradient flotation allowed for identification of two prostasome subpopulations with distinct size (56 ± 13 versus 105 ± 25 nm), but similar equilibrium buoyant density. The exosomal marker CD9 was present in both subpopulations, whereas GLI pathogenesis-related 2 was enriched in smaller prostasomes and Annexin A1 in larger prostasomes.

Density gradient flotation has also been employed to show that copurification of different types of EXOs occurs when using dUC only. Upon centrifugation, isolated EXOs (i.e., 100,000 × *g* pellets) float into an overlaid sucrose density gradient, which allows for fractionation (80, 84). Using this methodology, Bobrie et al. studied heterogeneity within EXOs derived from 4T1 mouse breast cancer cells (80). CD9 was found to be present in all sucrose fractions, with a strong enrichment in CD9 expressing EXOs present in specific low-density fractions (1.11 and 1.14 g/ml) and high-density fractions (1.26 and 1.29 g/ml). A similar pattern was observed for CD63. Mfge8, on the other hand, was enriched in EXOs present in the low-density fraction (1.14 g/ml). These differential levels of common EXO markers hint to the fact that secretion of distinct EXO subpopulation might occur.

Interestingly, inhibition of the small GTPase Rab27a in 4T1 tumor cells decreased CD63 levels in all fractions (i.e., to less than the detection limit), whereas levels of CD9 and Mfge8 were only moderately reduced Immuno electron microscopy allowed the authors to study the distribution of CD9 within the seemingly heterogeneous EXO populations in more detail. Similar to the flotation results, CD9 was observed in a variety of morphologically distinct EXO types (e.g., non cup-shaped and large cup-shaped). Rab27a inhibition resulted in a reduction of CD9 in larger EXOs only, providing further evidence for the fact that larger EXOs floating at 1.14 g/ml might require Rab27a for their secretion. Effects of Rab27a inhibition on the secretion of larger EVs recovered at 10,000 × *g* (i.e., MVs) were not observed. These findings clearly highlight heterogeneity in EV preparations isolated by UC and EVs in general.

Similar to findings by Bobrie et al., two distinct particle populations were observed after dUC-isolated EXOs (110,000 × *g* pellets) from B16F10 melanoma cells were subjected to upward flotation in a sucrose density gradient (87). One population was recovered from the lower density fractions, 1.12–1.19 g/ml (termed LD-EXO), while the second population associated with the higher density fractions 1.26–1.29 g/ml (termed HD-EXO). Transmission electron microscopy (TEM) and Nanoparticle Tracking Analysis were performed to determine the size distribution of the obtained subpopulations. The LD-EXO subpopulation was found to have a broader size range from 75 to 200 nm (majority peaking at 117 nm), whereas the HD-EXO subpopulations was found to be more homogenous in size and smaller than 100 nm (majority peaking at 66 nm). Proteomic analysis showed a strong enrichment of EV and EXO marker proteins ALIX, TSG101, CD9, CD81, and CD63 in LD-EXO and HD-EXO when compared with MVs (i.e., 10,000 × *g* pellets). Unique enrichment was observed for actinin alpha 4 (ACTN4) and cyclin Y (CCNY) in LD-EXO versus HD-EXO, and ephrin type-A receptor 2 (EPHA2) in HD-EXO versus LD-EXO. The RNA content of MVs was found to be similar to LD-EXO, although a larger contribution of small RNA to the total RNA pool was observed, whereas HD-EXO had a completely distinct RNA profile, e.g., lacking rRNA.

Although dUC and DG are the most commonly used techniques for EV isolation, their time-consuming nature, low and operator-dependent yield, and lack of automatization remain major drawbacks. On top of that it has been reported that EVs isolated with UC harbor lower functionality, most likely due to damaging forces exerted on the EVs during centrifugation at high speed (88). For this reason, multiple easy to use and more effective (i.e., higher yield and higher functionality) alternatives to these methods have been explored by researchers in the field.

### Size Exclusion Chromatography (SEC)

Size exclusion chromatography has emerged as a user-friendly alternative to dUC and DG. More importantly, SEC does not seem to impact EV integrity and as such it preserves EV functionality (88, 89). SEC is performed using a column containing small porous polymer beads (referred to as the stationary phase). As the particle-containing solution travels through the stationary phase, small particles are able to enter the porous beads. As a result, larger particles travel through the column more quickly than small particles and elute at an earlier time point than small particles. SEC has been widely utilized by researchers in the EV field, mainly as a straightforward method that overcomes problems associated with UC [e.g., vesicles disruption, aggregation, and co-purification of non-EV material (89, 90)]. More importantly, clear differences between functionality of dUC-isolated EVs (UC-EVs) and SEC isolated EVs (SEC-EVs) have been reported (88). TEM analysis of UC-EVs and SEC-EVs showed no major morphological differences (with both preparations containing large and sEVs), and Western blot analyses revealed that both UC-EVs and SEC-EVs were enriched for ALIX and CD63. However, a clear increase in EV-induced ERK1/2 phosphorylation in recipient endothelial cells was observed after treatment with SEC-EVs compared with UC-EVs, highlighting increased functionality of EVs isolated with SEC.

Through the appropriate choice of column resin, SEC may also be used to separate EV subpopulations from each other. Willms et al. utilized a custom SEC method (based on Sephacryl S-1000 resin) for the fractionation of UC-isolated EXOs according to size. On the basis of enrichment profiles of ACTN4, CCNY, and EPHA2, the presence of two distinct subpopulations of EXOs previously identified by sucrose DG was confirmed (82). Using a similar methodology, Willis et al. were able to separate EXO subtypes derived from human mesenchymal stem/stromal cells from bone marrow and Wharton’s jelly (91). EXOs were isolated by differential centrifugation, followed by tangential flow filtration and iodixanol density floatation, and subsequently fractionated by SEC. Large EXOs (>80 nm in size) were found to be enriched in CD63 and flotillin-1, whereas small EXOs (<80 nm in size) were enriched in ALIX and TSG101.

These findings further highlight the heterogeneity of EXO preparations, and the potential of SEC for isolation of specific EV and EXO subpopulations with increased functionality.

### Ultrafiltration (UF)

Ultrafiltration allows for separation using semipermeable membranes with defined molecular weight cutoffs or pore sizes. Particles with a molecular weight or size below the applied filter membrane pass through the membrane, whereas larger particles are retained. Similar to SEC, techniques based on UF may offer less harsh and straightforward alternatives to dUC, minimizing the exposure of EVs to high-centrifugal forces (92, 93).

Simpson et al. set out to reveal the presence of multiple EV subtypes released from LIM1863 colon cancer cells using a method based on a series of four filtration steps through hydrophilic PVDF membrane filters with different pore sizes (0.65, 0.45, 0.22, and 0.1 µm) (92). The obtained fractions, correlating to retained particles after filtration, were termed Fn1 (i.e., retentate after 0.65 µm filtration, subsequently pelleted by centrifugation 10,000 × *g*) to Fn5 (i.e. retentate after 0.1 µm filtration, subsequently pelleted by centrifugation 100,000 × *g*). Strong enrichment of ALIX, TSG101, and tetraspanins CD63 and CD81 was observed in Fn5. The first fraction (Fn1, obtained at 10,000 × *g*) lacked these markers. It was therefore concluded that fraction Fn5 contained EXOs and fraction Fn1 MVs. Fractions 2–4 were excluded for any further characterization due to low EV yield. Cryo-EM showed a homogenous distribution of particles in fraction Fn5, whereas Fn1 was heterogeneous in size containing a small percentage of very large EVs consistent with LOs as well. Based on dynamic light scattering, a similar heterogeneous pattern was observed for Fn1, while in Fn5 two distinct subsets of EXOs were observed, ranging in size from 50 to 80 and 120 to 200 nm in diameter, indicating the potential existence of EXO subpopulations. Subsequent proteomic analysis revealed 256 proteins common to both fractions, 98 unique proteins in the EXOs (i.e., Fn5), and 350 in the MVs (i.e., Fn1). EXOs were found to be enriched in common EXO marker proteins (TSG101, ALIX, and Syntenin) and a number of tetraspanins (e.g., CD9, CD63, CD82, CD151, TSPAN6, and TSPAN8) and integrins (ITGA2, ITGA6, ITG1, and ITGB4). MVs, on the other hand, were found to be selectively enriched in cytoskeletal elements (e.g., microtubules) and septins (along with their associated binding partners), and cytoskeleton associated proteins (e.g., Actin, Actinin, Dynamin, Myosins, Tubulin, and VDAC1/2).

Taken together, these findings provide further evidence for the unique composition of EV populations. UF allows for straightforward isolation of EV populations, although its limited options due to predefined membrane pore sizes is a major drawback.

### Asymmetrical-Flow Field-Flow Fractionation (A4F)

The experimental use of flow field flow fractionation (FFFF) was first described by Giddings et al. in 1966 (94, 95). In FFFF, particles are loaded into a small channel consisting of two semipermeable membranes, in which separation is based on the interaction of two flow streams (cross and longitudinal) with the particles. Differences in particle hydrodynamic diameter (i.e., size) and molecular weight result in differences in particle mobility through the channel, allowing for subsequent separation.

Asymmetrical-flow field-flow fractionation, a type of FFFF, is a relatively new technique to the EV field and makes use of a single semipermeable membrane. Only a small number of groups have reported using this technique to isolate and characterize EVs (92, 96–103). The main advantages of A4F over dUC are its mild character and its ability to separate materials over a wide colloidal size range. In other words, A4F could allow for fractionation of distinct EV populations (e.g., EXO, MV, and APO) and even EXO subpopulations, while preserving intact biophysical characteristics. In addition, there is reduced risk of potential loss of EVs due to adherence to membranes (UF) or a stationary phase (SEC). A4F does, however, require substantial experimental optimization for each application as there is no “one size fits all” setup.

Yang et al. utilized A4F for size fractionation of urinary EXOs from prostate cancer patients (104). The lipidomic profiles of obtained EXO fractions were investigated and compared with healthy controls. Urine was cleared by centrifugation at 12,000 × *g*, and EXOs were subsequently pelleted by centrifugation at 200,000 × *g*. Isolated EXOs were subjected to A4F, and five fractions were collected. An increase in the concentration of small EXOs was detected after fractionation of EXOs derived from the urine of prostate cancer patients, compared with healthy controls. CD9 was not detected in larger EXOs from both patients and controls (fraction 5), whereas ALIX was detected in fractions 2–4 for both groups, but not in fraction 5. On the basis of these findings, the authors decided to combine fractions 2 and 3 (F1; small EXOs < 150 nm) and fractions 4 and 5 (F2; large EXOs > 150 nm). Subsequently, lipidomic analysis of the two populations F1 and F2 was performed. In total, 286 lipids were identified from unfractionated EXOs of patients and healthy controls, with 270 of the identified lipids common to both groups. The authors were able to show that the levels of most lipid classes (except diaglycerol, triaglycerol, and cholesteryl ester) increased in patients with prostate cancer compared with healthy controls. This increase might originate from increased expression of fatty acid synthase in cancer cells. Interestingly, a number of lipid classes (phosphatidylcholine, phosphatidylethanolamine, phosphatidylethanolamine plasmalogen, phosphatidylserine, and SM) showed a higher increase in smaller EXOs, whereas all other classes did not show a size-dependent change. These findings might hint to the fact that smaller EXOs originate from different cells during prostate cancer progression.

Zhang et al. have recently shown that A4F allows for fractionation of EXOs, and isolation of EXO subpopulations based on size. Two distinct subpopulations of EXOs, Exo-L (large EXO vesicles, 90–120 nm) and Exo-S (small EXO vesicles, 60–80 nm), were isolated and characterized (105). In addition, the authors describe the discovery of a novel population of very small non-membranous nanoparticles (~35 nm), which they term exomeres (106).

The aforementioned findings clearly show that A4F can be utilized for isolation of EVs, and distinct EXO subpopulations. With the added benefit of avoiding exposure to external forces (e.g., high centrifugal speeds, interaction with resin) potentially influencing EV integrity.

### Microfluidic Isolation (MI)

Rapid advances made in the design and development of microfluidic systems have reached the EV field as well. The application of these devices, which allow precise control and manipulation of geometrically constrained fluids on a sub-millimeter scale, for EV isolation has been extensively reviewed by Gholizadeh et al. (107). Although the technique is not widely applied yet, Shin et al. highlight its potential for isolation of EV populations based on size (108). Based on TEM analysis and expression of the EXO marker syntenin, and the exclusion marker calreticulin, the authors were able to separate EXOs from large APOs. The very mild external forces, possibility to apply low sample volumes, and minimal sample dilution make MI a very attractive technique for EV isolation.

### Flow Cytometry (FC)

In contrast to aforementioned techniques, which are all based on size-dependent separation of EV populations, FC allows for separation based on EV surface composition. This technique has been widely used for counting and characterizing individual cells in heterogeneous populations of cells. In FC, particles are suspended in a hydrodynamically focused stream and passed through a series of lasers, and subsequent scattering by the particles is detected. Fluorescence (e.g., fluorescently labeled antibodies or fluorescent dyes) can be utilized to increase sensitivity and optimize detection of particles.

Its application for detection and potential sorting of EV populations is hampered mainly by their submicron size, complex composition, and low refractive index. Different FC techniques and the application of FC in the EV field have been extensively reviewed elsewhere (109).

Approaches which allow for detection of EVs and distinct subpopulations have been published (106, 110). In short, Nolte-’t Hoen et al. combined general PKH67 labeling of EVs isolated by dUC (100,000 × *g* pellets), with selective labeling with fluorescently labeled antibodies (e.g., R-PE- or APC-labeled). This approach allowed for discrimination between T-cell- and DC-derived EXOs. A double labeling approach with MHC II and MFG-E8 even allowed for detection of subtle changes in the composition of EVs derived from LPS stimulated DCs, highlighting the potential strength of this technique for detection of EV subpopulations. Recent progress has been made in further optimization of FC approaches (e.g., varying nozzle size and sheath pressure), and subsequent particle analysis. Groot Kormelink et al. described the development of a method for high-resolution FC-based particle quantification, characterization, and sorting (110). Potential pitfalls for single EV analysis and sorting (e.g., coincidence and swarm effects) were addressed. EVs were isolated from bone marrow-derived mast cells using dUC (100,000 × *g*) and general CFSE EV labeling was combined with R-PE-labeled CD63 and APC-labeled CD9 antibodies. Utilizing their optimized method, the authors were able to accurately separate and sort CD63 positive EVs from CD9-positive EVs. This highlights the potential application of FC for sorting EV populations and distinct subpopulations. A similar method for analysis and sorting termed fluorescence-activated vesicle sorting was published by Higginbotham et al. (111). EVs were isolated from a human colorectal cancer (DiFi) cell line by dUC (165,000 × *g* pellets). Subsequent fluorescent detection using Alexa-488 and Alexa-467 conjugated EGFR and CD9 antibodies allowed for the identification of individual EVs. Utilizing this approach in combination with an antibody specific to the activated conformation of EGFR, distinct EV subpopulations with expression of conformationally active EGFR were detected.

### Immunocapture (IC)

Recent advances in FC approaches in combination with immune affinity capture have resulted in the development of novel multiplex bead-based platforms, allowing analysis of the surface protein composition of EVs (112). This new sandwich method is based on the use of a set of up to 39 different types of beads, each coupled to a single capture antibody, combined with fluorescently labeled detection antibodies, and allows the analysis of EVs that carry surface markers recognized by both antibodies. This multiplex platform enables an easy screening of surface markers on subpopulations of EVs. Combining this multiplex platform with stimulated emission depletion, a super-resolution microscopy technique that bypasses the diffraction limit of light microscopy, makes it possible to visualize individual EVs and detection of protein markers on single EVs. The use of these technologies allowed the detection of heterogeneous distribution of tetraspanins on EVs, and the identification of distinct EV subpopulations produced by specific cell types. For instance, NK cell-derived EVs were found to be devoid of tetraspanin CD9 but express CD63 and CD81, while platelet-derived EVs express abundant CD9 and CD63, but lack CD81. In addition, different discrete subpopulations of EVs derived from activated B lymphocytes were identified, based on the relative expression of B cell markers CD19 and CD20 and activation markers CD80 and CD86. Therefore, employment of this novel technology based on immunoaffinity isolation allows the identification and physical separation of different EV subpopulations from a heterogeneous mixture.

The combination of these techniques allows for very rapid and broad screening of EV samples, but physical binding of the EVs to beads hampers the study of EV functionality. The technique could, however, be employed to deplete EV samples of all EVs expressing a specific marker (i.e., deplete of all CD9-bearing EVs), which would allow for the study of differences in functional effects of EV subpopulations. Taken together, these methods are very powerful techniques, which are easy to use, quick, and can even be performed without extensive prior purification steps. Further optimization of flow cytometers will lower detection limits and improve resolution.

### Perspectives

Great advances have been made in the development of techniques for the isolation of EVs, which will allow for further dissection of EV heterogeneity, and further elucidation of EV biogenesis and functions. To date, UC remains the most commonly used technique for isolation of EVs. However, multiple techniques commonly used in other fields (e.g., SEC and FC) have now been successfully optimized for isolation and characterization of EVs. Each technique has its own specific advantages (e.g., high resolution and/or purity) and drawbacks (e.g., time-consuming and/or expensive), as summarized in Table 1. Selection of the most suitable technology therefore highly depends on the research question and definition of EV subtype (size versus marker expression).

**Table 1:** Advantages and disadvantages of isolation techniques.

	dUC	UF	DG	SEC	FC	IC	A4F	MI
Sample purity	med.	low	high	high	high	high	high	high
Ability to resolve subpopulations	low	med.	med.	med.	high	high	med.	med.
Ease of use	mod.	easy	dif.	easy	mod.	mod.	dif.	mod.
Time	long	short	long	inter.	inter.	inter.	inter.	inter.
Scalable	no	yes	no	yes	yes	yes	yes	yes
Possibility to automate	dif.	dif.	dif.	mod.	mod.	mod.	dif.	mod.
Downstream application	char. and func.	char. and func.	char. and func.	char. and func.	char. only	char. only	char. and func.	char. and func.
Cost	med.	low	med.	med.	high	high	high	med.

*dUC, differential ultracentrifugation; UF, ultrafiltration; DG, density gradient; SEC, size exclusion chromatography; FC, flow cytometry; IC, immunocapture; A4F, asymmetrical flow field flow fractionation; MI, microfluidic isolation; med., medium; mod., moderate; dif., difficult; char., characterization; func., functionality*.

Sample purity—Are obtained samples free of contaminating proteins, lipids, and fragments? *Ability to resolve subpopulations*—Is the method able to isolate homogenous populations, and as such suitable for population studies? *Ease of use*—Is the method easy to use, are special training or equipment needed? *Time*—Is the method time-consuming? *Scalable*—Is the isolation process scalable? *Possibility to automate*—Is it possible to automate the isolation process? *Downstream application*—Is it possible to use the obtained samples for functional assays (i.e., *in vitro* and *in vivo*)? *Cost*—What are the costs associated with method of isolation (e.g., specialized equipment, time, and consumables)?

## EV Populations and Their Role in Cancer

The established role of tumor-derived EVs in cancer has been widely reviewed elsewhere (113–115). We will here focus on reported functional differences between tumor EV populations based on size (i.e., LOs, APOs, MVs, and EXOs, including recent emerging evidence on EXO heterogeneity and the role of specific EXO subpopulations), and between EV populations based on surface composition (i.e., integrin and tetraspanin expression).

It has long been speculated that differences in the composition of EV populations could result in differences in biological functions (15). Studying these differences has, however, been hampered by the lack of specific markers for the different EV populations released by tumor and other cells. As highlighted in the Section “Techniques for Isolation of EV Populations,” current methods of EV isolation and methods of fractionation often result in mixtures of EV populations. Nevertheless, there is increasing evidence regarding the link between EV type and biological function in cancer.

### Subpopulations Based on EV Size

#### APOs, MVs, and EXOs

Differences between the roles of commonly reported EV populations (i.e., APOs, MVs, and EXOs) in cancer have been evaluated in a number of studies. Proteomic analysis of EXOs and MVs derived from SH-SY5Y neuroblastoma cells highlight the differences in composition between these major EV populations (116). For this study, EVs were isolated by ultracentrifugation (100,000 × *g*) and subsequently purified by iodixanol DG. A homogenous EXO population (30–100 nm in diameter) highly enriched in exosomal markers TSG101 and ALIX was retrieved from low-density fractions of the gradient, whereas MVs (>200 nm in diameter) were retrieved from higher density fractions (with low TSG101 and ALIX expression). EXOs were enriched with ESCRT components (e.g., VPS24, VPS32, and VPS36), tetraspanins (e.g., CD81, TSPAN9, and TSPAN14), annexins (ANXA7), flotillins, and integrins (ITGA3). MVs, on the other hand, were found to be enriched in Rac GTPase-activating protein 1, protein disulfide-isomerase A3, spectrin beta chain, non-erythrocytic 2, Mucin-19, 3 ubiquitin-protein ligase, keratin, type I cytoskeletal 18 (KRT18), kinesin-like protein 14, kinesin-like protein 4A, vimentin, 40S ribosomal protein S9, 40S ribosomal protein S18, and matrix metalloproteinase-2 (MMP2). On top of that, CD81 and MMP2 were exclusively found in, respectively, EXOs and MVs, suggesting their potential use as *bona fide* markers for these EV populations. Functional enrichment analysis hinted to the fact that EXOs and MVs might function through different biological pathways. EXOs were found to be enriched in signaling proteins (e.g., proteins implicated in ESCRT, syndecan signaling, and membrane trafficking), whereas MVs were found to be enriched in enzymes and proteins implicated in gene expression and translation. Comparison of the identified MV and EXO content against COSMIC (Catalogue of Somatic Mutations in Cancer) and EST (Expressed Sequence Tag) databases revealed that proteins exclusively identified in EXO were highly abundant in most cancer types, whereas exclusive MV content was found to be less common in other types of cancer. Upon evaluation of functional effects, SH-SY5Y EXOs were indeed found to stimulate SK-N-BE2 cell *in vitro* proliferation and cell migration to a higher extent compared with MVs.

On the contrary, it has been reported that MVs isolated from LIM1863 colon cancer cells induce higher invasive activity in recipient fibroblasts cells (NIH3T3 cells) than EXOs (117). Based on proteomic enrichment of invasion, migration, and motility-related components in the EXOs and MVs, the authors argued that these EV populations might stimulate invasion through different mechanisms. However, the difference in tumor cell source and isolation technique (UF versus dUC) may have in part accounted for the observed difference in functionality of MVs and EXOs.

Investigations into the procoagulant and immunogenic properties of B16-F1 melanoma EXOs, MVs, and APOs provide an interesting body of evidence on the functionality of these populations in cancer (118). Fibrin and thrombin generation assays revealed a higher coagulative potential of APOs compared with MVs and EXOs, with EXOs being the least coagulative population. Evaluation of the immunogenic potential of the populations revealed that APOs offer protection against *in vivo* B16 tumor formation and could therefore play a major role in anti-cancer immunity. Thrombotic events are a major cause of death in cancer patients and EVs have been shown to be involved in thrombotic events (119). This finding may therefore have implications when taking into account that anti-cancer therapy (e.g., cytoablative therapy) might promote release of APOs, in that way increasing the risk of thrombotic events, while at the same time stimulating an anti-cancer immune response.

#### Large Oncosomes

It has recently been shown that prostate cancer (LNCaP)-derived LOs affect the surrounding stroma cells, establishing a positive feedback loop affecting tumorigenesis and tumor progression (120). The authors established that active EV-associated Akt1, found to be enriched in prostate cancer-derived LOs compared with EXOs, is a key player both *in vitro* and *in vivo*. *In vitro*, conditioned medium of normal prostate associated fibroblasts (NAFs) treated with LOs induced a significant increase in tube formation of endothelial cells (HUVECs), compared with EXO-treated NAFs. Alterations in expression of factors (e.g., interleukin-6, MMP1, MMP9, α-smooth muscle actin, TGFb1, and thrombospondin 1) in fibroblasts treated with LOs reflected a pro-vascularization phenotype. Moreover, *ex vivo* treatment of NAFs with LOs before combined *in vivo* injection with DU145 (human prostate cancer cells) resulted in significantly enhanced tumor growth compared with injection of DU145 alone. Stimulation of MYC-dependent transcription/processes was found to mediate the observed fibroblast reprogramming. Importantly, inhibition of LO uptake through interference with active endocytic pathways prevented the observed effects. Proteomic analysis of LO revealed enrichment of proteins involved in metabolic processes relevant to cancer, including the metabolic enzyme aspartate transaminase (GOT1). LOs were able to transfer GOT1 and increase glutamate production in recipient cells, suggesting an effect of LOs on metabolic functions in cancer cells. Furthermore, it has been shown that LOs mediate transfer of miR-1227, influencing migration of cancer-associated fibroblasts (CaFs) (75).

#### EXO Subpopulations

Aforementioned observations provide evidence for functional differences between the major established EV populations. Increasing evidence, however, highlights heterogeneity within these secreted populations, particularly within EXOs, the smallest population of EVs. For example, findings by Willms et al. show that B16F10 melanoma cells release two distinct subpopulations of EXOs (i.e., LD-EXO and HD-EXO) with unique biological effects (87). Treatment of recipient endothelial cells with LD-EXO resulted in changes in the expression of 257 genes, compared with 1,116 genes after treatment with HD-EXO, when compared with PBS treatments. GO statistical enrichment analysis on the genes affected by LD-EXO and HD-EXO revealed a number of differentially significantly affected protein classes (e.g., PANTHER protein classes “G-protein modulator,” GO molecular function “small GTPase regulator activity,” GO biological process “DNA replication,” and GO cellular component “protein–DNA complex”). In addition, a strong upregulation of the glutamine transporter SLC38A1 was observed after treatment with the larger EXOs (LD-EXO). Minciacchi et al. previously reported a similar finding, observing alterations in glutamine metabolism after treatment with large EVs (73).

These observations suggest that cancer cells, on top of classic EV populations, release distinct EXO subpopulations which may exert unique biological functions.

### Subpopulations Based on EV Surface Composition

The EV field has made great progress defining major EV populations secreted by cells, mainly based on their underlying biogenesis and biophysical characteristics. Nevertheless, overlap in size (as illustrated in Figure 1) and lack of *bona fide* makers hamper the study of EV heterogeneity. An updated categorization of EVs based on enrichment of tetraspanins, one of the most commonly found protein class in EVs, has recently been put forward (81). Interestingly, integrins and other adhesion receptors are consistently found among the proteins associated with tetraspanins in tetraspanin-enriched microdomains (TEMs), regardless of the cell type under consideration (121–123). Through these interactions, tetraspanins exert an important regulatory control on the adhesive and signaling capacities of associated adhesion molecules. Alterations in tetraspanin and integrin content could thus result in unique functional roles of EV subpopulations.

#### Tetraspanins and Integrins in the Targeting and Uptake of EVs

Targeting and uptake of EVs by recipient cells are processes still poorly understood, accumulating evidence, however, suggests that the interplay between tetraspanins, integrins, and other adhesion molecules within the context of TEMs, in both the EV and the target cell membranes, is fundamental in the regulation of these processes. For instance, the tetraspanin CD9 has been shown to regulate the adhesive activity of immunoglobulin superfamily adhesion molecules activated leukocyte cell adhesion molecule (ALCAM) (124), intercellular adhesion molecule 1 (ICAM-1), vascular cell adhesion protein 1 (VCAM-1) (125, 126), as well as that of β1 and β3 integrins (122, 123, 127, 128) and, more recently, of the leukocyte β2 integrin LFA-1 (129). Other tetraspanins, including CD63, CD81, and CD151, also associate with distinct integrins and other adhesion receptors, exerting regulatory effects on their activities (122, 126, 130). Accordingly, it comes as no surprise that most of the antibodies that have been shown so far to interfere with EV binding and uptake by recipient cells are directed against tetraspanins (CD9, CD81, Tspan8, and CD151), integrins (CD11a, CD11b, CD11c, CD18, CD49c, CD49e, CD51, and CD61), or integrin counter-receptors (CD54 = ICAM-1, CD106 = VCAM-1), which are expressed either on the surface of EVs or on the plasma membrane of target cells (32, 131, 132).

Extracellular vesicle tetraspanins and integrins have been shown to play a pivotal role in directing the targeting and selective uptake of EVs by recipient cells. EVs produced by the highly metastatic rat pancreatic adenocarcinoma cell line BSp73ASML preferentially target lung fibroblasts and lymph node stroma cells, triggering in these cells the upregulation or *de novo* expression of several adhesion molecules, chemokines, growth factors, and proteases, thus promoting pre-metastatic niche formation. By contrast, EVs differing only in the expression of Tspan8 (released from the Tspan8-transfected non-metastatic variant BSp73AS-Tspan8 cell line), rather selectively target endothelial cells (133). The additional transfection of integrin β4 in BSp73AS-Tspan8 cells increases their metastatic capacity, and the EVs derived from these cells are no longer targeted to endothelial cells but show a preferential binding and incorporation into stromal cells, accumulating in the liver and lung after their intravenous injection (134).

Metastasis occurs in an organ-specific pattern, and it has become evident that EVs play a key role in directing organotropism (135). There is a growing body of evidence emerging which shows that specific tumors produce distinct types of EVs that facilitate the organ-specific metastasis. Hoshino et al. were able to show that organotropism is directed by EV integrins, with tumor-derived EVs being capable of redirecting metastasis of tumor cells which normally lack metastatic capacity. EV integrins α6β4 and α6β1 were found to favor binding to lung-resident fibroblasts and epithelial cells, governing the lung metastasis tropism, while EV integrin αvβ5 dictates binding of EVs to Kupffer cells and associates with liver metastasis. The authors also proposed that EV integrins not only mediate the adhesion of EVs but also trigger signaling and inflammatory responses in target cells, rendering the organ permissive for the growth of metastatic cells. Importantly, packaging of integrins into EVs seems to be a selective process, since EV integrin expression did not reflect cellular expression.

Findings by Yue et al. provide more *in vivo* evidence for the link between EV composition and biological functions (134), in this case the specific involvement of EV tetraspanins CD151 and Tspan8, two major metastasis-promoting tetraspanins that play a role in metastasis formation in several tumor systems. The authors were able to show that knockdown of CD151 and Tspan8 resulted in loss of metastatic capacity of rat pancreatic adenocarcinoma tumor cells, which was regained after pre-treatment with EVs (dUC 100,000 × *g*) derived from highly metastatic wild-type pancreatic cells. Cross talk between tumor-derived EVs and the matrix is strongly influenced by EV tetraspanins (CD151 and Tspan8), mainly due to their associations with integrins (ITGA3, ITGB4, and ITGAM) and proteases (MMP2, MMP9, MMP14, and CD13), which facilitate binding, motility, and matrix degradation. In addition, EVs can affect hematopoietic and stroma cell activation including (lymph)angiogenesis and promote EMT in neighboring non-metastatic tumor cells.

Furthermore, it has recently been reported that neuroblastoma cells secrete different EXO subpopulations which differ in their cargoes and target different cells, namely neurons or glial cells (136). These two subpopulations of EXOs can be distinguished by the mutually exclusive presence of tetraspanin CD63 and amyloid precursor protein (APP). While CD63-containing EXOs indifferently bind to neurons and glial cells, APP-containing EXOs bind specifically to and can be endocytosed by neurons. The generation of these distinct EXO subpopulations seems to depend on sorting inside ILVs, with APP sorting depending on ESCRT, while CD63 sorting into ILVs seems to be ESCRT independent. Therefore, this study provides further support for the concept that the selective targeting (and thus function) of different exosomal subpopulations produced by tumor cells may be dictated by the presence or absence of a particular tetraspanin.

#### Roles of Tetraspanins and Integrins in the Immunostimulatory and Immunosuppressive Properties of EV Subpopulations in Cancer

A large part of the immunosuppression observed in neoplastic lesions has been proposed to be primarily mediated by EVs released from tumors (137). Cancer cells secrete large numbers of EVs with the potential to establish a communication network by interacting and delivering their cargo to a multitude of recipient cells, including CaFs, epithelial, endothelial, and immune cells (138). The overall effect of such a network is partly mediated by the balance between the EV-induced stimulatory and suppressive effects on cancer immunity, although the latter seem to prevail and the net result is the promotion of tumor progression and dissemination. Although this topic has recently been covered in detail by several excellent reviews (138, 139), we here provide a brief summary of the EV-mediated interplay between tumor and immune cells. On the immunostimulatory side, tumor cell-derived EVs can induce antitumor immune responses by transferring tumor-specific antigens to DCs, which then induce potent CD8^+^ T cell-dependent antitumor effects (140–142). These findings have led to investigating the use of cancer-derived EVs as antitumoral vaccines (143, 144). In addition, tumor-derived EVs can also stimulate antitumor immunity through the activation of macrophages and NK cells (32).

On the other hand, EVs exert a number of immunosuppresive effects in cancer hosts, including impairment of DC differentiation, maturation, and function, polarization toward tumor-promoting macrophages, decrease of NK and CTL proliferation and cytotoxicity, and induction of regulatory T and B cells [reviewed in Ref. (137, 145, 146)]. In some cases, the specific EV components responsible for the immunosuppressive effects have been identified (138).

To induce any of these types of immune cell dysfunction and immunosupression, a required initial step is the interaction of EVs with the relevant target immune cells which, as discussed above, is a receptor-mediated process that can trigger intracellular signaling and subsequent cellular responses. This initial interaction with immune cells can be followed by the release of their content into the cell cytoplasm either by direct fusion of their membranes or by uptake into an endosomal compartment (followed by fusion with its membrane) through one or more of a variety of mechanisms, as reviewed in Ref. (131, 132). Depending on the particular type of recipient immune cell, this uptake mechanism may involve macropinocytosis, phagocytosis, caveolin-mediated, clathrin-mediated, or lipid-raft-mediated endocytosis. For instance, antigen-presenting cells such as DCs and macrophages display a high phagocytic activity which represents the main mechanism responsible for the uptake and internalization EVs. By contrast, T lymphocytes do not seem to be very efficient in internalizing EVs, and the predominant mechanism for delivering their message into these cells is mediated through their interaction with cellular surface proteins that trigger intracellular signaling and provoke the T cell response (35, 147).

Tetraspanins, either directly or through their associated proteins in TEMs, such as integrins and Ig-SF (immunoglobulin superfamily) molecules, have been reported to play essential roles in ligand-mediated interactions, as well as in fusion and endocytosis phenomena, which highlights their potential as important mediators in tumor-induced immunosuppression. In this regard, numerous studies have demonstrated that tetraspanins CD9 and CD81 are pivotal in membrane fusion processes, including fertilization (oocyte-sperm fusion), skeletal myotube formation, fusion of macrophages to form multinucleated osteoclasts or giant cells, and enveloped virus entry and egress (148, 149). Other tetraspanins, such as Tspan-13/NET-6, have also been implicated in some of these fusion processes (150). It is also well established that different tetraspanins (CD151, CD63, and CD81) play a role in infections by cytomegalo- and papillomaviruses through regulation of the endocytosis of viral particles (151).

Through the use of specific antibodies, a role in the targeting and uptake of EXOs by DCs has been demonstrated for tetraspanins CD9 and CD81 and integrin α_vβ3_ on the exosomal membrane, and integrin LFA-1 (α_Lβ2_) and ICAM-1 (CD54) on the recipient DC membrane. Also, as discussed above, another tetraspanin, Tspan8, in complex with integrin subunit CD49d (α4), has been shown to mediate the selective binding and uptake of tumor EXOs by endothelial cells, leading to their activation and proliferation (148).

Taken together, these studies point to a potential general role of tetraspanins and associated integrins in the binding, fusion, and/or uptake of EVs by recipient cells. Furthermore, changes in just the expression of a specific tetraspanin or integrin can completely alter the selective targeting and uptake of EXOs by recipient cells, and their subsequent functional effects. As such tetraspanin and integrin profile could serve as a basis to categorize EV subpopulations.

## Conclusion and Perspectives

Findings on the role of EV populations in cancer have clearly indicated the diverse biological functions of EVs. However, current limitations of isolation and characterization techniques remain to hamper addressing heterogeneity of biophysical characteristics and composition of secreted EVs and secreted EV populations (i.e., subpopulations). Nevertheless, EV heterogeneity is currently one of the major challenges that has to be addressed by the EV field. Advances in isolation and separation techniques will allow for more in depth and precise studies on the underlying complexity of EV biogenesis. One important consideration that researchers have to take into account is how to correctly make direct functional comparisons between populations, mainly due to challenges in the precise quantification and characterization of EV populations with different biophysical and molecular composition, which hampers subsequent normalization (18). Nevertheless, great advances have been made in understanding the heterogeneous nature of secreted EVs, and defining EV subpopulations. Continuous research will allow for further elucidation of the precise role of EVs in both physiological and pathophysiological processes, ultimately accelerating development of EVs as therapeutics and diagnostics.

## Author Contributions

EW, CC, and PV conceived and wrote the manuscript. EW and IM designed the figures. IM and MW commented and edited the manuscript. All authors read and approved the final manuscript.

## Conflict of Interest Statement

MW is founder and non-executive director of, and consultant to Evox Therapeutics. All other authors declare that the research was conducted in the absence of any commercial or financial relationships that could be construed as a potential conflict of interest.
